# The Effect of Transcranial Direct Current Stimulation on Jaw Motor Function Is Task Dependent: Speech, Syllable Repetition and Chewing

**DOI:** 10.3389/fnhum.2018.00033

**Published:** 2018-02-13

**Authors:** Meg Simione, Felipe Fregni, Jordan R. Green

**Affiliations:** ^1^Department of Pediatrics, MassGeneral Hospital for Children, Boston, MA, United States; ^2^Spaulding-Labuschagne Neuromodulation Center, Spaulding Rehabilitation Hospital, Boston, MA, United States; ^3^Speech and Feeding Disorders Laboratory, MGH Institute of Health Professions, Boston, MA, United States

**Keywords:** transcranial direct current stimulation, mandible, task-dependency, speech, chewing, biomechanics

## Abstract

Motor cortex transcranial direct current stimulation (tDCS) has been shown to enhance motor learning in healthy adults as well as various neurological conditions. However, there has been limited data on whether tDCS enhances jaw motor performance during different oral behaviors such as speech, maximum syllable repetition, and chewing. Because the effects of anodal and cathodal stimulation are known to be dependent on task demands, we hypothesized that tDCS would have a distinct effect on the jaw motor performance during these disparate oral behaviors. Ten healthy adults completed speech, maximum syllable repetition, and chewing tasks as their jaw movements were recorded using 3D optical motion capture during sham, anodal, and cathodal tDCS. Our findings showed that compared to the sham condition, jaw displacements during speech and syllable repetition were smaller during anodal stimulation, but larger during cathodal stimulation for syllable repetition and chewing indicating improved performance during anodal tDCS. On the other hand, there were no effects of anodal tDCS during chewing. These results confirm our hypotheses that: (1) tDCS induces a significant effect on jaw motor function; (2) its effects are polarity dependent; and (3) its effects are dependent on the task demands on jaw motor function. These findings support future studies exploring the effects of tDCS on persons with oral sensorimotor impairments and the development of therapeutic protocols.

## Introduction

Transcranial direct current stimulation (tDCS) is a form of non-invasive brain stimulation that is widely being tested to enhance motor learning (Bolognini et al., 2009; Grimaldi et al., 2016). Although spoken word is highly dependent on motor performance, the impact of tDCS on speech motor performance has rarely been investigated (Fiori et al., 2014; Bashir and Howell, 2017; Chesters et al., 2017). When using tDCS, low amplitude currents pass through the scalp and underlying cortical tissues from the anode electrode to the cathode electrode. This current flow has a differential effect on resting-state membrane potentials of cortical neurons depending on the polarity of the stimulation (Fregni and Pascual-Leone, 2007) with anodal stimulation increasing neuronal excitability and cathodal stimulation decreasing neuronal excitability (Nitsche and Paulus, 2000). Polarity effects have been most consistently observed in studies on motor cortex stimulation (Nitsche and Paulus, 2000; Nitsche et al., 2003; Boggio et al., 2006; Vines et al., 2006; Cogiamanian et al., 2007; Tanaka et al., 2009; Stagg et al., 2011; Krishnan et al., 2014) that have reported anodal excitatory effects (i.e., increased motor evoked potentials, increased muscle activity, endurance and force) and cathodal inhibitory effects (i.e., decreased motor evoked potentials, degraded performance).

The effects of anodal or cathodal tDCS on plasticity however, is complex and highly dependent on task-specific neural activity and behavioral demands (Bikson and Rahman, 2013; Miniussi et al., 2013; Pirulli et al., 2014; Bestmann et al., 2015). Given that a motor behavior is a dynamic process and engages different neural areas according to the complexity of the task, thus, the effects of tDCS depends on the task and relative neural engagement. For complex behaviors, such as spoken word production, this entails that stimulation of the motor cortex is likely to affect the many concurrent processes (i.e., linguistic, cognitive, attention) engaged during speech production (Bohland et al., 2010) and level of skill required for the specific task. Given these complex interactions between polarity and task demands, it is critical to investigate the specific effects of tDCS on speech motor performance, as to optimize the outcomes of tDCS therapeutic trials (Bikson and Rahman, 2013).

In this exploratory study, we investigate if tDCS has short-term effects on jaw motor function during three oral motor behaviors—speech, maximum syllable repetition, and chewing gum. Because these three tasks have different behavioral goals and physiologic demands (Moore et al., 1988; Smith and Denny, 1990; Moore, 1993), neural modulation might be expected to have a distinct effect on the neural networks that govern these disparate oral behaviors. Speech engages motor and linguistic neural networks (Guenther, 2006; Ackermann and Riecker, 2010; Price, 2012) whereas chewing predominantly engages sensorimotor networks that involve motor cortex and brainstem central pattern generators (Lund, 1991; Quintero et al., 2013). Chewing gum is presumably highly automatized (Lund and Kolta, 2006; Mistry and Hamdy, 2008), and lacks linguistic demands that influence jaw movements produced during speech and maximum syllable repetition tasks. Unlike chewing, the maximum syllable repetition task engages speech motor networks but is likely to only minimally activate linguistic networks that are engaged during meaningful speech (Ziegler, 2002; Bohland and Guenther, 2006; Sörös et al., 2006; Kent, 2015). This task, however, might be motorically and cognitively more demanding as it involves more effort than speech or chewing to produce syllables as fast as possible. We use Levelt’s “mental syllabary” (Levelt et al., 1999) to guide our interpretation of the effects of tDCS and how that might affect the access of precompiled speech units resulting in changes to motor performance.

Our empirical framework assumes that enhanced jaw motor function during these tasks will be manifested as an economization of effort (i.e., an efficient jaw movement strategy) whereas disrupted jaw motor function will be evidenced by increased effort (i.e., an inefficient jaw movement strategy). This framework is consistent with limb studies, that have demonstrated that larger and faster movements tend to be more energetically costly (Oliveira et al., 2005); and when motivated, persons converge rapidly on economical and accurate biomechanic strategies rapidly (Manohar et al., 2015). The adaptation of efficient jaw movement strategies is well documented during fast speech where most talkers achieve rapid rates by minimizing jaw displacement rather than increasing jaw speed (Nelson et al., 1984; Mefferd and Green, 2010). This economization of effort is learned during the first decade of life as children gradually acquire adult-like rates of speech by minimizing the extent of articulatory movements rather than increasing the speed (Nip and Green, 2013). By contrast, large, inefficient jaw excursions have been observed to be a characteristic of impaired speech in some neurologically impaired populations (Nip, 2013).

Within this framework, given that anodal tDCS is particularly useful for novel learning (or learning after a brain lesion), it is conceivable that anodal tDCS worsens performance of highly learned and simple movements (such as chewing gum) while cathodal tDCS has the opposite effect. It is also conceivable that anodal tDCS would lead to motor performance improvements in more complex motor tasks that can be enhanced with additional learning. To test these hypotheses, healthy adults completed speech, maximum syllable repetition, and chewing tasks while their jaw movements were recorded using 3D optical motion capture during sham, anodal and cathodal tDCS.

## Materials and Methods

The research study was conducted at the Speech and Feeding Disorders Lab at the Massachusetts General Hospital (MGH) Institute of Health Professions and all participants gave informed written consent in accordance with the Declaration of Helsinki. The experimental protocol was approved by The Spaulding Rehabilitation Hospital Institutional Review Board.

Ten adults (6 females; 4 males) between the ages of 18–45 years participated in the study. Their primary language was English and they had no history of speech, language, hearing, or neurological disorders or contraindications to tDCS including metal in the head, implanted brain medical device, or seizures. All participants passed a pure tone audiometric screening at 1, 2 and 4 kHz at 30 dB. The participants attended three sessions that were separated by a minimum of 48 h. The sessions consisted of one of three possible tDCS conditions: anodal, cathodal, or sham. The order of each condition was counterbalanced across participants.

### Experimental Tasks

During each session, participants completed three tasks: speech, syllable repetition, and chewing gum. The tasks were selected to represent a range of tasks with differing linguistic and motor demands. For the speech task, the participants were instructed to repeat a phrase commonly used in motor speech studies, “Buy Bobby a puppy”, 12 times at their normal speaking rate (Nip and Green, 2013). For the syllable repetition task, participants were instructed to produce the syllable, /ba/, as fast as possible on one breath. For the chewing task, participants were given a sized controlled piece of gum and instructed to chew on their right molars 12 times at their natural rate.

**Figure 1:**
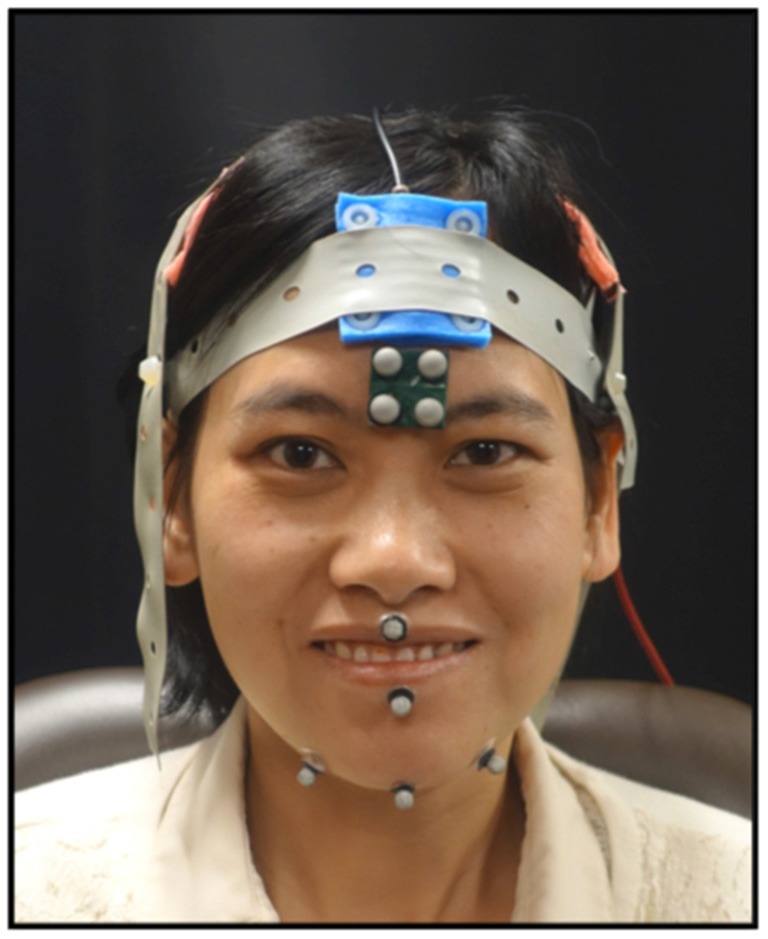
Shown here is marker placement used to record mandibular movements and the electrode montage for the transcranial direct current stimulation (tDCS). Informed written consent was obtained for the use of this image for publication.

As the participants completed the tasks, jaw movements were recorded using 3D optical motion capture. An eight-camera system was used to record jaw movements at 120 frames per second (Motion Analysis Corporation, Santa Rosa, CA, USA). Three reflective spherical markers were adhered to the jaw—one marker was placed on the jaw gnathion and two markers were placed to the right and left of the jaw gnathion (see Figure 1). The marker to the right of the jaw gnathion was used for analysis as it is less prone to error due to flesh movements (Green et al., 2007). One marker from the rigid 4-marker array affixed to the forehead was selected to subtract head movements from the jaw movement trajectories. Full-face videos and audio data were recorded and synchronized with the motion capture data at the time of the collection.

### Transcranial Direct Current Stimulation

While completing the tasks, the participants received one of three possible tDCS conditions (i.e., anodal, cathodal, or sham). A tDCS machine (Soterix Medical) was used to deliver 2 mA of current to the scalp electrodes (5 cm × 5 cm) in a 2 × 1 montage. The active electrodes were placed bilaterally over the sensorimotor cortex. We used a bilateral montage because of the bilateral innervation of the mandible. For placement, the 10–20 EEG system was used and the electrodes were placed 2 cm down and over from C3/C4 (see Figure 1). The reference electrode was placed over the midline frontal area. The duration of the stimulation was 20 min and all tasks were completed during active stimulation. Sham stimulation consisted of ramping up for 15 s immediately followed by ramping down of the current. The participants and the experimenter were blinded to the tDCS conditions.

### Data Analysis

The tasks were parsed using the distance signal, full-face recordings and audio. The distance signals were digitally low-pass filtered at a cutoff frequency of 10 Hz (Butterworth, eight pole). The Euclidian distance between the top head marker and the right jaw marker was calculated and used for all analyses (see Figure 1). From the phrase, the second syllable, /ba/, was parsed. For the syllable repetition task, 12 syllables across the entire sequence were selected at regularly spaced intervals. For the chewing task, 12 chewing cycles were used. After identifying the syllables and chewing cycles, the closing stroke of the movement was parsed. The first and last closing stroke were then excluded and the remaining 10 trials were used for analyses. For some trials, the chewing task had only nine cycles as some cycles were deemed to be extraneous jaw movements related to managing the bolus. The criteria for determining a chewing cycle was based on the video recordings and the velocity signal. To be accepted as a chewing cycle, the velocity of the individual cycle had to fall within the 80th percentile of the velocity of the entire sequence. For each closing stroke (i.e., maximum oral opening to maximum oral closure), the duration, range and peak speed of mandibular movement were calculated using SMASH, a custom MATLAB program (Green et al., 2013).

### Statistical Analysis

Descriptive statistics were calculated by averaging the means of all the participants for each dependent variable. A linear mixed model was constructed for each of the dependent variables to determine the effects of polarity for each task. The experimental condition (i.e., anodal tDCS, cathodal tDCS and sham) was used as the fixed factor and the participants were the random factor. A multiple comparison of means (Tukey Contrasts) was used to test for differences between the three experimental conditions. Cohen’s *d* effect sizes were also calculated between the sham and the two experimental conditions (i.e., sham—anodal; sham—cathodal). Statistical analysis was completed using R (R Core Team, 2016).

## Results

**Figure 2:**
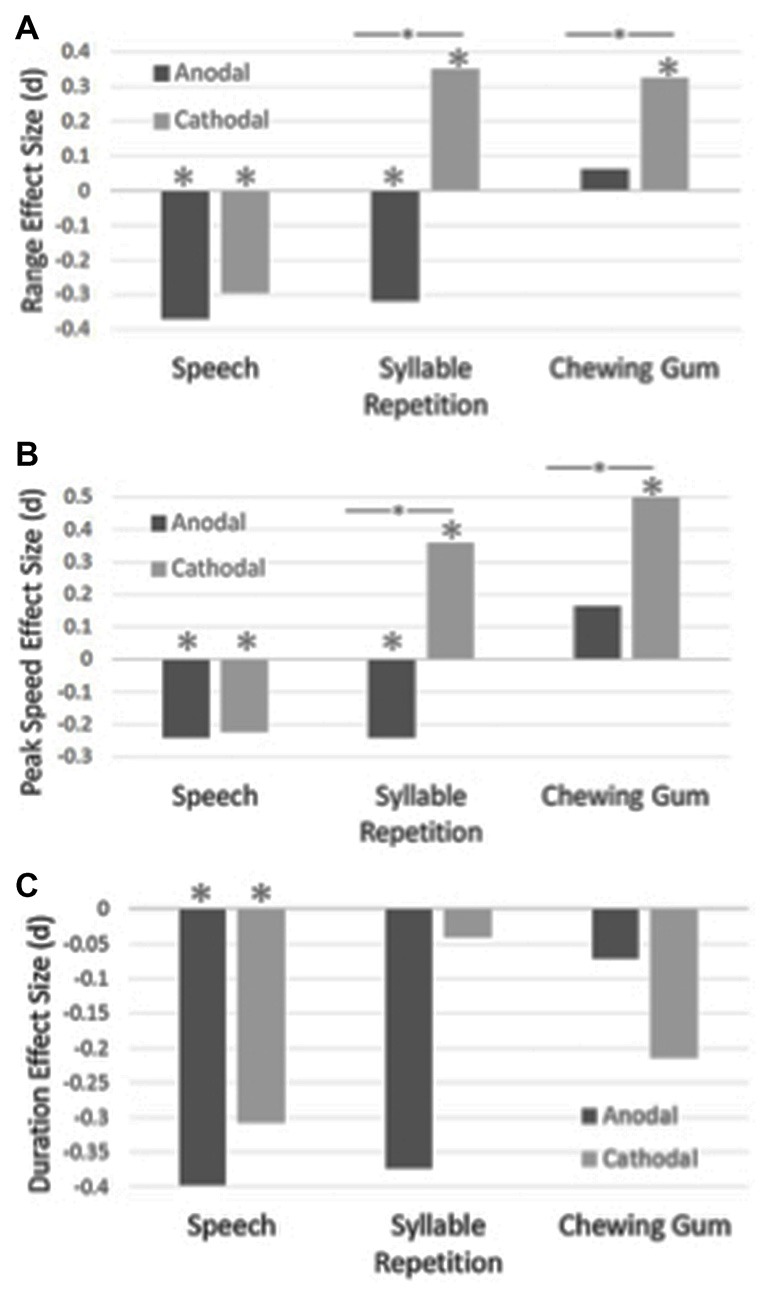
The effect sizes for range of mandibular movements are shown in Panel **(A)**, peak mandibular speed are shown in Panel **(B)** and duration of the closing stroke are shown in Panel **(C)**, for the speech, syllable repetition and chewing tasks. The effect sizes were calculated between the sham and anodal/cathodal conditions. Statistically significant differences are shown with an asterisk. Statistically significant differences between the anodal and cathodal conditions are shown with a bar and asterisk.

Table 1 shows the means and standard deviations for the experimental conditions. The effect sizes for duration, range of movement and peak mandibular speed for the three tasks are shown in Figure 2.

**Table 1:** Task descriptive statistics for the experimental conditions.

Task	tDCS Condition	Range of movement (mm) M (SD)	Peak speed (mm/s) M (SD)	Duration (s) M (SD)
Speech				
	Sham	4.67 (1.47)	67.44 (19.44)	0.13 (0.03)
	Anodal	4.14 (1.42)	62.58 (20.79)	0.12 (0.02)
	Cathodal	4.23 (1.51)	63.16 (19.16)	0.12 (0.03)
Syllable repetition				
	Sham	2.64 (0.85)	46.37 (15.11)	0.10 (0.01)
	Anodal	2.35 (0.97)	42.45 (17.43)	0.09 (0.01)
	Cathodal	2.97 (1.07)	52.58 (19.32)	0.10 (0.01)
Chewing gum				
	Sham	6.35 (1.45)	45.33 (14.44)	0.34 (0.05)
	Anodal	6.43 (1.13)	47.93 (17.34)	0.33 (0.06)
	Cathodal	6.78 (1.25)	54.48 (21.56)	0.32 (0.09)

### Speech

A main effect of condition was found for the speech task for duration, *F*_(289,2)_ = 8.00, *p* < 0.001, range, *F*_(289,2)_ = 11.34, *p* < 0.001 and peak mandibular speed, *F*_(289,2)_ = 7.78, *p* < 0.001. *Post hoc* analysis revealed that duration became shorter, as compared to sham, with anodal tDCS, *p* < 0.001, and cathodal tDCS, *p* = 0.003. Range of movement became smaller with anodal tDCS, *p* < 0.001, and cathodal tDCS, *p* < 0.001, as compared to sham. Peak speed also became slower as compared to the sham condition for the anodal, *p* < 0.001, and the cathodal condition, *p* = 0.004.

### Syllable Repetition

A main effect of condition was found for the syllable repetition task for range, *F*_(289,2)_ = 18.76, *p* < 0.001 and peak speed, *F*_(289,2)_ = 19.61, *p* < 0.001. No main effect was found for duration, *F*_(289,2)_ = 1.95, *p* < 0.14. Jaw movements became larger with cathodal stimulation as compared to the sham, *p* = 0.003, and anodal condition, *p* < 0.001, but the range became smaller with anodal stimulation, *p* = 0.01. With cathodal stimulation, peak speed was faster than the sham condition, *p* < 0.001, and the anodal condition, *p* < 0.001. Jaw movements were slower with anodal stimulation, *p* = 0.04.

### Chewing Gum

For the chewing task, a main effect of condition was found for range, *F*_(288,2)_ = 4.50, *p* = 0.01 and peak speed, *F*_(288,2)_ = 14.29, *p* < 0.001. No main effect was found for duration, *F*_(288,2)_ = 1.47, *p* = 0.23. The range of jaw movements became larger with cathodal stimulation as compared to sham, *p* = 0.01, and anodal stimulation, *p* = 0.056. With cathodal stimulation, peak speeds became faster as compared to the sham, *p* < 0.001, and anodal conditions, *p* < 0.001.

## Discussion

In this exploratory study, we investigated if tDCS affects jaw motor function during speech, maximum syllable repetition, and chewing. Because these behaviors have distinct physiological demands and behavioral goals, we hypothesized that the effects of neural modulation on jaw motor function would be task-dependent. Our findings suggest that the interaction of polarity and task demands resulted in biomechanically efficient jaw movements (i.e., smaller movement displacements) during some tasks while jaw movements during other tasks became biomechanically inefficient (i.e., larger movement displacements). We speculate that the differential effects for the three tasks may be due to either motor drive or extramotor effects depending on the behavior.

### Anodal Stimulation Resulted in Improved Performance for Speech

During anodal stimulation, jaw movements became smaller and slower and duration became shorter relative to the sham condition. This finding is indicative of increased biomechanic efficiency. By reducing their jaw excursions, talkers were economizing their articulatory efforts (Perkell et al., 1997), which is consistent with the articulatory strategy identified by Lindblom (1990) as hypo-articulation. This bias towards minimizing movements is also seen in limb studies as an energy minimization strategy (Oliveira et al., 2005).

We speculate that the speech changes likely resulted from an effect that was not only from the motor cortex, but also that tDCS may have enhanced connected speech related areas, such as, the cognitive and linguistic processes that support speech rather than the isolated modulation of the primary motor cortex (Jacobson et al., 2012). For example, anodal stimulation may have facilitated cognitive control processes that allowed participants to select the most efficient movement strategy. Talkers were able to consider the cost of minimizing articulator movements while still achieving an accurate acoustic production of the phrase. The increased cognitive effort would presumably have a top-down effect on jaw motor function (Sarter et al., 2006). Previous studies have shown that tDCS can enhance cognitive functioning (Fregni et al., 2005; Coffman et al., 2014) and in this study, those processes may have been modulated via network-level effects (Polanía et al., 2011). Several studies have shown that anodal tDCS leads to an enhancement of cognitive tasks the subjects are engaged in by accessing other networks that can improve performance (Lapenta et al., 2013).

Anodal stimulation may have also impacted linguistic networks. Previous tDCS studies have shown improvements in language processing in healthy adults, such as picture naming and verbal fluency (Iyer et al., 2005; Cattaneo et al., 2011; Holland et al., 2011; Meinzer et al., 2016; Lifshitz-Ben-Basat and Mashal, 2017). But few studies have focused on motor speech production in healthy adults (Fiori et al., 2014; Bashir and Howell, 2017; Chesters et al., 2017). Fiori et al. (2014) found shorter durations when participants repeated tongue twisters with anodal stimulation to the left frontal cortex, which was consistent with our findings for the speech task. Potential mechanisms of linguistic enhancement include Levelt’s “mental syllabary” (Levelt et al., 1999). The mental syllabary is a hypothesized cortical center (Riecker et al., 2008; Brendel et al., 2011) that stores precompiled speech units that are accessed during speech. The syllabary allows for the rapid retrieval of commonly produced and overlearned syllables. Therefore, tDCS may be improving access and retrieval to the syllabary that then allows for more efficient articulations. For example, many studies have demonstrated that familiar words and words with higher phonotactic probability are produced with shorter durations than unfamiliar words and words with low phonotactic probabilities (Wright, 1979; Munson, 2001; Munson et al., 2005) illustrating a strong word-frequency effect purportedly due to the rapid access of the phonological code (Levelt, 1999).

### Cathodal Stimulation Resulted in Inefficient Jaw Movements for Chewing

Contrary to the speech task, during chewing, cathodal stimulation had the effect of making jaw movements less efficient. We speculate that the differential tDCS effects on chewing as compared to speech may be due to the different demands resulting in task-specific induced neural activity. The inhibitory release of motor drive or extramotor processes may be responsible for these changes to jaw motor function. Previous studies have shown that cathodal tDCS over the motor cortex decreases the excitability of inhibitory circuits (Batsikadze et al., 2013; Sasaki et al., 2016). This release of inhibition of motor drive is consistent with the large movements and faster speeds found during the chewing task.

Another possible explanation may be that cathodal stimulation suppressed relevant sensory signals resulting in degraded performance. Although chewing is heavily mediated by central pattern generators that are responsible for the rhythmic patterns of chewing, it is also dependent on the sensorimotor cortex to provide sensory feedback control regarding the changes to the physical properties of food during the masticatory sequence (Lund, 1991; Mistry and Hamdy, 2008). Cathodal stimulation may have led to a decrease in neuronal activity in the pathways necessary for chewing. Lang et al. (2005) found that cathodal tDCS over the motor cortex led to decreased regional cerebral blood flow in the cortex. This inhibition may have resulted in less efficient movements evidenced in the chewing task. Although in this study we cannot ascertain the reasons why anodal tDCS did not result in changes to jaw motor function during the chewing task, it is possible that that chewing task was too simple and thus there were no additional possibilities for performance enhancement resulting in a ceiling effect for anodal stimulation. This effect has been observed during cognitive tasks; while anodal tDCS enhances performance of more complex tasks, it does not in simpler tasks (Woods et al., 2016). In this context, we can conclude that the results of tDCS are not only highly dependent on the nature of the task, but on the task performance of the person being stimulated. For example, if a person had never chewed gum before then they may have benefited from tDCS.

### Anodal and Cathodal Stimulation Resulted in Differential Effects for Maximum Syllable Repetition

Unlike speech and chewing, polarity effects were found for the syllable repetition task. Relative to sham, jaw movements became smaller and slower with anodal stimulation and larger and faster with cathodal stimulation. During anodal stimulation, talkers economized their articulatory efforts while producing the syllable /ba/ as fast as possible. Similar to speech, the efficient jaw movements observed during anodal stimulation may be explained by extramotor processes. That is, talkers may have selected the most efficient movement control strategies due to enhancement of cognitive process (Manohar et al., 2015) or they may have been able to retrive syllables via the mental syllabary faster (Levelt et al., 1999).

During cathodal stimulation, jaw movements became larger, which is not an optimal strategy for this task. Similar to chewing, either the inhibitory release of motor drive or attenuation of relevant neural signals may explain jaw performance during syllable repetition. Cathodal stimulation during the rapid syllable task did not result in the same truncation of jaw movements that was observed during speech. The syllable repetition task resulted in differential polarity findings whereas cathodal and anodal stimulation resulted in similar findings for the speech task (i.e., smaller jaw displacements). Arguably the speech task was more linguistically complex, but the syllable repetition task involved more motoric and cognitive (i.e., attention and intention) demands. A meta-analytical review found that differential effects between anodal and cathodal stimulation were not consistently found in language studies (Jacobson et al., 2012). In another meta-analytical review of language-based studies, they concluded that additional studies are needed to determine if there are dissociable effects between polarities (Price et al., 2015). We speculate that these inconsistent effects (i.e., between anodal and cathodal for the speech task and between speech and syllable repetition tasks) may be due to the differing task demands across oral behaviors.

### Limitations

This study presented with some limitations. Because our speaking task was a simple sentence repetition, it remains unknown how tDCS affects jaw control during more demanding speaking conditions. Because this study was exploratory, we speculated on the possible causes (i.e., motor drive or extramotor processes) of the differential findings. Also, due to the exploratory nature of this study, our sample size was small therefore we must be cautious in concluding the null effects for the chewing task and the duration variable. Functional imaging would have provided additional information to elucidate the underlying mechanisms of change due to tDCS. Also, the bilateral, bi-cephalic placement that was used in this study due to the bilateral innervation of the jaw has not been widely used therefore it remain unknown how that montage affected the findings as compared to other studies.

## Conclusion

The purpose of this study was to investigate the effects of tDCS on jaw motor function during speech, maximum syllable repetition, and chewing gum. Our findings revealed that anodal stimulation resulted in an efficient movement strategy for speech and syllable repetition whereas cathodal stimulation resulted in a less efficient movement strategy for syllable repetition and chewing. The results suggest that the differential effects were an interaction of polarity and distinct behavioral demands for each task. We posit that the effects for some tasks were due to motor drive, while the effects for other tasks were due to network-level extramotor effects particularly in tasks where there was room for improvement. In fact, results in patients with motor speech deficits may be likely larger. The results of this study help to understand polarity and task demands during different oral behaviors for the development of future therapeutic protocols for persons with oral sensorimotor impairments.

## Author Contributions

MS, FF and JRG all made substantial contributions to the conception, design and the interpretation of data for the work. MS and JRG made contributions to acquisition and analysis of the work. MS and JRG drafted the work and FF revised it for critically important intellectual content. MS, FF and JRG all gave final approval of the version to be published and agree to be accountable for all aspects of the work in ensuring that questions related to the accuracy or integrity of any part of the work are appropriately investigated and resolved.

## Conflict of Interest Statement

The authors declare that the research was conducted in the absence of any commercial or financial relationships that could be construed as a potential conflict of interest. The reviewer SK declared a shared affiliation, though no other collaboration, with one of the authors MS to the handling Editor, who ensured that the process nevertheless met the standards of a fair and objective review.

## References

[B1] Ackermann H. Riecker A. (2010). “Cerebral control of motor aspects of speech production: neurophysiological and functional imaging data,” in Speech Motor Control: New Developments in Basic and Applied Research, eds Maassen B. van Lieshout P. (Oxford: Oxford University Press), 117–134.

[B2] Bashir N. Howell P. (2017). P198 tDCS stimulation of the left inferior frontal gyrus in a connected speech task with fluent speakers. Clin. Neurophysiol. 128:e111 10.1016/j.clinph.2016.10.317

[B3] Batsikadze G. Moliadze V. Paulus W. Kuo M.-F. Nitsche M. A. (2013). Partially non-linear stimulation intensity-dependent effects of direct current stimulation on motor cortex excitability in humans. J. Physiol. 5917, 1987–2000. 10.1113/jphysiol.2012.249730 23339180 PMC3624864

[B4] Bestmann S. de Berker A. O. Bonaiuto J. (2015). Understanding the behavioural consequences of noninvasive brain stimulation. Trends Cogn. Sci. 19, 13–20. 10.1016/j.tics.2014.10.003 25467129

[B5] Bikson M. Rahman A. (2013). Origins of specificity during tDCS: anatomical, activity-selective, and input-bias mechanisms. Front. Hum. Neurosci. 7:688. 10.3389/fnhum.2013.00688 24155708 PMC3800813

[B6] Boggio P. S. Castro L. O. Savagim E. A. Braite R. Cruz V. C. Rocha R. R.. (2006). Enhancement of non-dominant hand motor function by anodal transcranial direct current stimulation. Neurosci. Lett. 404, 232–236. 10.1016/j.neulet.2006.05.051 16808997

[B8] Bohland J. W. Bullock D. Guenther F. H. (2010). Neural representations and mechanisms for the performance of simple speech sequences. J. Cogn. Neurosci. 22, 1504–1529. 10.1162/jocn.2009.21306 19583476 PMC2937837

[B7] Bohland J. W. Guenther F. H. (2006). An fMRI investigation of syllable sequence production. Neuroimage 32, 821–841. 10.1016/j.neuroimage.2006.04.173 16730195

[B9] Bolognini N. Pascual-Leone A. Fregni F. (2009). Using non-invasive brain stimulation to augment motor training-induced plasticity. J. Neuroeng. Rehabil. 6:8. 10.1186/1743-0003-6-8 19292910 PMC2667408

[B10] Brendel B. Erb M. Riecker A. Grodd W. Ackermann H. Ziegler W. (2011). Do we have a “mental syllabary” in the brain? An fMRI study. Motor Control 15, 34–51. 10.1123/mcj.15.1.34 21339513

[B11] Cattaneo Z. Pisoni A. Papagno C. (2011). Transcranial direct current stimulation over Broca’s region improves phonemic and semantic fluency in healthy individuals. Neuroscience 183, 64–70. 10.1016/j.neuroscience.2011.03.058 21477637

[B12] Chesters J. Hsu J. H. Bishop D. V. Watkins K. E. Mottonen R. (2017). Comparing effectiveness of three TDCS protocols on online and offline components of speech motor learning. Brain Stimul. 10, e27–e28. 10.1016/j.brs.2017.04.029

[B13] Coffman B. A. Clark V. P. Parasuraman R. (2014). Battery powered thought: enhancement of attention, learning, and memory in healthy adults using transcranial direct current stimulation. Neuroimage 85, 895–908. 10.1016/j.neuroimage.2013.07.083 23933040

[B14] Cogiamanian F. Marceglia S. Ardolino G. Barbieri S. Priori A. (2007). Improved isometric force endurance after transcranial direct current stimulation over the human motor cortical areas. Eur. J. Neurosci. 26, 242–249. 10.1111/j.1460-9568.2007.05633.x 17614951

[B15] Fiori V. Cipollari S. Caltagirone C. Marangolo P. (2014). “If two witches would watch two watches, which witch would watch which watch?” tDCS over the left frontal region modulates tongue twister repetition in healthy subjects. Neuroscience 256, 195–200. 10.1016/j.neuroscience.2013.10.048 24184977

[B17] Fregni F. Boggio P. S. Nitsche M. Bermpohl F. Antal A. Feredoes E.. (2005). Anodal transcranial direct current stimulation of prefrontal cortex enhances working memory. Exp. Brain Res. 166, 23–30. 10.1007/s00221-005-2334-6 15999258

[B16] Fregni F. Pascual-Leone A. (2007). Technology insight: noninvasive brain stimulation in neurology—perspectives on the therapeutic potential of rTMS and tDCS. Nat. Clin. Pract. Neurol. 3, 383–393. 10.1038/ncpneuro0530 17611487

[B18] Green J. R. Wang J. Wilson D. L. (2013). “SMASH: a tool for articulatory data processing and analysis,” in Interspeech 1331–1335. Available online at: http://www.isca-speech.org/archive/interspeech_2013/i13_1331.html

[B19] Green J. R. Wilson E. M. Wang Y.-T. Moore C. A. (2007). Estimating mandibular motion based on chin surface targets during speech. J. Speech Lang. Hear. Res. 50, 928–939. 10.1044/1092-4388(2007/066) 17675597 PMC2745713

[B20] Grimaldi G. Argyropoulos G. P. Bastian A. Cortes M. Davis N. J. Edwards D. J.. (2016). Cerebellar transcranial direct current stimulation (ctDCS): a novel approach to understanding cerebellar function in health and disease. Neuroscientist 22, 83–97. 10.1177/1073858414559409 25406224 PMC4712385

[B21] Guenther F. H. (2006). Cortical interactions underlying the production of speech sounds. J. Commun. Disord. 39, 350–365. 10.1016/j.jcomdis.2006.06.013 16887139

[B22] Holland R. Leff A. P. Josephs O. Galea J. M. Desikan M. Price C. J.. (2011). Speech facilitation by left inferior frontal cortex stimulation. Curr. Biol. 21, 1403–1407. 10.1016/j.cub.2011.07.021 21820308 PMC3315006

[B23] Iyer M. B. Mattu U. Grafman J. Lomarev M. Sato S. Wassermann E. M. (2005). Safety and cognitive effect of frontal DC brain polarization in healthy individuals. Neurology 64, 872–875. 10.1212/01.wnl.0000152986.07469.e9 15753425

[B24] Jacobson L. Koslowsky M. Lavidor M. (2012). TDCS polarity effects in motor and cognitive domains: a meta-analytical review. Exp. Brain Res. 216, 1–10. 10.1007/s00221-011-2891-9 21989847

[B25] Kent R. D. (2015). Nonspeech oral movements and oral motor disorders: a narrative review. Am. J. Speech Lang. Pathol. 24, 763–789. 10.1044/2015_AJSLP-14-0179 26126128 PMC4698470

[B26] Krishnan C. Ranganathan R. Kantak S. S. Dhaher Y. Y. Rymer W. Z. (2014). Anodal transcranial direct current stimulation alters elbow flexor muscle recruitment strategies. Brain Stimul. 7, 443–450. 10.1016/j.brs.2014.01.057 24582369

[B27] Lang N. Siebner H. R. Ward N. S. Lee L. Nitsche M. A. Paulus W.. (2005). How does transcranial DC stimulation of the primary motor cortex alter regional neuronal activity in the human brain? Eur. J. Neurosci. 22, 495–504. 10.1111/j.1460-9568.2005.04233.x 16045502 PMC3717512

[B28] Lapenta O. M. Minati L. Fregni F. Boggio P. S. Miniussi C. Nitsche M. A. (2013). Je pense donc je fais: transcranial direct current stimulation modulates brain oscillations associated with motor imagery and movement observation. Front. Hum. Neurosci. 7:256. 10.3389/fnhum.2013.00256 23761755 PMC3674333

[B29] Levelt W. J. M. (1999). Models of word production. Trends Cogn. Sci. 3, 223–232. 10.1016/s1364-6613(99)01319-4 10354575

[B30] Levelt W. J. Roelofs A. Meyer A. S. (1999). A theory of lexical access in speech production. Behav. Brain Sci. 22, 1–38; discussion 38–75. 10.1017/S0140525X99001776 11301520

[B31] Lifshitz-Ben-Basat A. Mashal N. (2017). Improving naming abilities among healthy young-old adults using transcranial direct current stimulation. J. Psycholinguist. Res. [Epub ahead of print]. 10.1007/s10936-017-9516-9 28856553

[B32] Lindblom B. (1990). “Explaining phonetic variation: a sketch of the H&H theory,” in Speech Production and Speech Modelling, eds Hardcastle W. J. Marchal A. (Netherlands: Springer), 403–439.

[B33] Lund J. P. (1991). Mastication and its control by the brain stem. Crit. Rev. Oral Biol. Med. 2, 33–64. 10.1177/10454411910020010401 1912143

[B34] Lund J. P. Kolta A. (2006). Generation of the central masticatory pattern and its modification by sensory feedback. Dysphagia 21, 167–174. 10.1007/s00455-006-9027-6 16897322

[B35] Manohar S. G. Chong T. T. J. Apps M. A. J. Batla A. Stamelou M. Jarman P. R.. (2015). Reward pays the cost of noise reduction in motor and cognitive control. Curr. Biol. 25, 1707–1716. 10.1016/j.cub.2015.05.038 26096975 PMC4557747

[B36] Mefferd A. S. Green J. R. (2010). Articulatory-to-acoustic relations in response to speaking rate and loudness manipulations. J. Speech Lang. Hear. Res. 53, 1206–1219. 10.1044/1092-4388(2010/09-0083) 20699341 PMC3548454

[B37] Meinzer M. Yetim Ö. McMahon K. de Zubicaray G. (2016). Brain mechanisms of semantic interference in spoken word production: an anodal transcranial Direct Current Stimulation (atDCS) study. Brain Lang. 157–158, 72–80. 10.1016/j.bandl.2016.04.003 27180210

[B38] Miniussi C. Harris J. A. Ruzzoli M. (2013). Modelling non-invasive brain stimulation in cognitive neuroscience. Neurosci. Biobehav. Rev. 37, 1702–1712. 10.1016/j.neubiorev.2013.06.014 23827785

[B39] Mistry S. Hamdy S. (2008). Neural control of feeding and swallowing. Phys. Med. Rehabil. Clin. N. Am. 19, 709–728. 10.1016/j.pmr.2008.05.002 18940637

[B40] Moore C. A. (1993). Symmetry of mandibular muscle activity as an index of coordinative strategy. J. Speech Hear. Res. 36, 1145–1157. 10.1044/jshr.3606.1145 8114481 PMC3984287

[B41] Moore C. A. Smith A. Ringel R. L. (1988). Task-specific organization of activity in human jaw muscles. J. Speech Hear. Res. 31, 670–680. 10.1044/jshr.3104.670 3230897

[B42] Munson B. (2001). Phonological pattern frequency and speech production in adults and children. J. Speech Lang. Hear. Res. 44, 778–792. 10.1044/1092-4388(2001/061) 11521771

[B43] Munson B. Swenson C. L. Manthei S. C. (2005). Lexical and phonological organization in children evidence from repetition tasks. J. Speech Lang. Hear. Res. 48, 108–124. 10.1044/1092-4388(2005/009) 15938063

[B44] Nelson W. L. Perkell J. S. Westbury J. R. (1984). Mandible movements during increasingly rapid articulations of single syllables: preliminary observations. J. Acoust. Soc. Am. 75, 945–951. 10.1121/1.390559 6707325

[B45] Nip I. S. B. (2013). Kinematic characteristics of speaking rate in individuals with cerebral palsy: a preliminary study. J. Med. Speech Lang. Pathol. 20, 88–94. 25364223 PMC4213862

[B46] Nip I. S. B. Green J. R. (2013). Increases in cognitive and linguistic processing primarily account for increases in speaking rate with age. Child Dev. 84, 1324–1337. 10.1111/cdev.12052 23331100 PMC3633670

[B47] Nitsche M. A. Paulus W. (2000). Excitability changes induced in the human motor cortex by weak transcranial direct current stimulation. J. Physiol. 527, 633–639. 10.1111/j.1469-7793.2000.t01-1-00633.x 10990547 PMC2270099

[B48] Nitsche M. A. Schauenburg A. Lang N. Liebetanz D. Exner C. Paulus W.. (2003). Facilitation of implicit motor learning by weak transcranial direct current stimulation of the primary motor cortex in the human. J. Cogn. Neurosci. 15, 619–626. 10.1162/089892903321662994 12803972

[B49] Oliveira F. T. P. Elliott D. Goodman D. (2005). Energy-minimization bias: compensating for intrinsic influence of energy-minimization mechanisms. Motor Control 9, 101–114. 10.1123/mcj.9.1.101 15784952

[B50] Perkell J. Matthies M. Lane H. Guenther F. Wilhelms-Tricarico R. Wozniak J. (1997). Speech motor control: acoustic goals, saturation feedback and internal models effects, auditory. Speech Commun. 22, 227–250. 10.1016/s0167-6393(97)00026-5

[B51] Pirulli C. Fertonani A. Miniussi C. (2014). Is neural hyperpolarization by cathodal stimulation always detrimental at the behavioral level? Front. Behav. Neurosci. 8:226. 10.3389/fnbeh.2014.00226 25018709 PMC4073198

[B52] Polanía R. Nitsche M. A. Paulus W. (2011). Modulating functional connectivity patterns and topological functional organization of the human brain with transcranial direct current stimulation. Hum. Brain Mapp. 32, 1236–1249. 10.1002/hbm.21104 20607750 PMC6870160

[B54] Price C. J. (2012). A review and synthesis of the first 20 years of PET and fMRI studies of heard speech, spoken language and reading. Neuroimage 62, 816–847. 10.1016/j.neuroimage.2012.04.062 22584224 PMC3398395

[B53] Price A. R. McAdams H. Grossman M. Hamilton R. H. (2015). A meta-analysis of transcranial direct current stimulation studies examining the reliability of effects on language measures. Brain Stimul. 8, 1093–1100. 10.1016/j.brs.2015.06.013 26210573 PMC4833093

[B55] Quintero A. Ichesco E. Myers C. Schutt R. Gerstner G. E. (2013). Brain activity and human unilateral chewing: an fMRI study. J. Dent. Res. 92, 136–142. 10.1177/0022034512466265 23103631 PMC3545687

[B56] R Core Team (2016). R: A Language and Environment for Statistical Computing. Vienna, Austria Available online at: https://www.R-project.org/

[B57] Riecker A. Brendel B. Ziegler W. Erb M. Ackermann H. (2008). The influence of syllable onset complexity and syllable frequency on speech motor control. Brain Lang. 107, 102–113. 10.1016/j.bandl.2008.01.008 18294683

[B58] Sarter M. Gehring W. J. Kozak R. (2006). More attention must be paid: the neurobiology of attentional effort. Brain Res. Rev. 51, 145–160. 10.1016/j.brainresrev.2005.11.002 16530842

[B59] Sasaki R. Miyaguchi S. Kotan S. Kojima S. Kirimoto H. Onishi H.. (2016). Modulation of cortical inhibitory circuits after cathodal transcranial direct current stimulation over the primary motor cortex. Front. Hum. Neurosci. 10:30. 10.3389/fnhum.2016.00030 26869909 PMC4740366

[B60] Smith A. Denny M. (1990). High-frequency oscillations as indicators of neural control mechanisms in human respiration, mastication and speech. J. Neurophysiol. 63, 745–758. 10.1152/jn.1990.63.4.745 2341873

[B61] Sörös P. Sokoloff L. G. Bose A. McIntosh A. R. Graham S. J. Stuss D. T. (2006). Clustered functional MRI of overt speech production. Neuroimage 32, 376–387. 10.1016/j.neuroimage.2006.02.046 16631384

[B62] Stagg C. J. Jayaram G. Pastor D. Kincses Z. T. Matthews P. M. Johansen-Berg H. (2011). Polarity and timing-dependent effects of transcranial direct current stimulation in explicit motor learning. Neuropsychologia 49, 800–804. 10.1016/j.neuropsychologia.2011.02.009 21335013 PMC3083512

[B63] Tanaka S. Hanakawa T. Honda M. Watanabe K. (2009). Enhancement of pinch force in the lower leg by anodal transcranial direct current stimulation. Exp. Brain Res. 196, 459–465. 10.1007/s00221-009-1863-9 19479243 PMC2700246

[B64] Vines B. W. Nair D. G. Schlaug G. (2006). Contralateral and ipsilateral motor effects after transcranial direct current stimulation. Neuroreport 17, 671–674. 10.1097/00001756-200604240-00023 16603933

[B65] Woods A. J. Antal A. Bikson M. Boggio P. S. Brunoni A. R. Celnik P.. (2016). A technical guide to tDCS and related non-invasive brain stimulation tools. Clin. Neurophysiol. 127, 1031–1048. 10.1016/j.clinph.2015.11.012 26652115 PMC4747791

[B66] Wright C. E. (1979). Duration differences between rare and common words and their implications for the interpretation of word frequency effects. Mem. Cognit. 7, 411–419. 10.3758/bf03198257 542114

[B67] Ziegler W. (2002). Task-related factors in oral motor control: speech and oral diadochokinesis in dysarthria and apraxia of speech. Brain Lang. 80, 556–575. 10.1006/brln.2001.2614 11896657

